# Grain-Free Diets for Dogs and Cats: An Updated Review Focusing on Nutritional Effects and Health Considerations

**DOI:** 10.3390/ani15142020

**Published:** 2025-07-09

**Authors:** Jing Zhang, Yun Ji, Ying Yang, Zhenlong Wu

**Affiliations:** 1 State Key Laboratory of Animal Nutrition and Feeding, Department of Companion Animal Science, China Agricultural University, Beijing 100193, China; s20233040807@cau.edu.cn (J.Z.); jean500@163.com (Y.J.); cauvet@163.com (Y.Y.); 2 Beijing Jingwa Agricultural Science and Technology Innovation Center, #1, Yuda Road, Pinggu, Beijing 101200, China

**Keywords:** grain-free diets, pet food, dog, cat, nutrition, health

## Abstract

In recent years, grain-free pet food has gained increasing attention due to its widespread promotion as a natural and hypoallergenic diet. This trend has prompted us to conduct an in-depth critical assessment of the nutritional implications of such diets. This review systematically analyzed the nutritional components of commercially available grain-free pet food, revealing significant variability in the nutritional composition of commercial pet diets. Moreover, grain-free formulations typically exhibit higher protein and fat levels. On the other hand, this review further explores the multifaceted health impacts of these diets on pets, including digestive function, cardiovascular health, allergic reactions, blood sugar regulation, mycotoxin safety, and palatability. This review points out that grain-free diets may offer potential benefits, such as improved blood sugar control, reduced mycotoxin exposure, and enhanced palatability; however, they also pose clear risks, particularly the potential association with dilated cardiomyopathy (DCM) in dogs. Additionally, the presence or absence of grains in the diet does not mitigate the risk of allergies. A grain-free diet is only intended to help specific individuals (such as those who are sensitive to grains/gluten) avoid allergens. Ultimately, the review emphasizes that nutritional adequacy, balance, and meeting individualized needs are the cornerstones of pet health, aiming to provide valuable insights for pet caregivers, veterinarians, and researchers.

## Simple Summary

Currently, there is a significant amount of controversy surrounding the advantages and disadvantages of feeding pets grain-free foods. This review provides an analysis of the nutritional composition of currently available grain-free pet foods on the market and evaluates their influence on pet health and welfare. Drawing upon existing scientific evidence, this review explores the multifaceted implications of grain-free diets on various aspects of companion animals, including gastrointestinal health, cardiovascular health, allergy, glycemic regulation, mycotoxin safety, and palatability for pets. With a focus on both canine and feline nutrition, this review aims to better assist pet caregivers in raising their pets in a scientific, economic, and efficient manner.

## 1. Introduction

Commercial pet foods were introduced as convenient and nutritionally balanced alternatives to homemade diets in the early 20th century. Over the past decades, the population of companion animals has increased, and there are 370 million pet cats and 470 million dogs kept as pets worldwide [1]. According to a global pet survey conducted in 2024, 63% of respondents reported owning pets, with 86% of these caregivers keeping dogs and 58% keeping cats [2]. Furthermore, the pet food market has continued to grow steadily. In Europe, FEDIAF’s latest annual report indicates that pet food constitutes 44.2% of the industry’s total value [3]. Similarly, China’s pet food market accounted for 52.8% of its companion animal industry value in 2024 [4]. These parallel figures demonstrate the pivotal role of pet food across whole markets. Over time, there has been a paradigm shift in the perception and treatment of pets, with many pet caregivers now considering their animals as integral members of their families. Consequently, there is an increasing demand for superior quality and more natural diets for pets [5]. As the pet food industry expanded, various functional ingredients with specific effects on health have been included in the pet food to enhance taste, texture, and nutritional profiles. Simultaneously, these ingredients increase the marketability of the product and attract greater interest from caregivers. Grain-free diets for pets have garnered increasing attention in recent years due to the perception that grains included in the food may confer potential adverse effects to dogs and cats. A report from the Food and Drug Administration (FDA) demonstrated that the intake of grain-free pet food was positively correlated with DCM in dogs [6], thereby fueling concerns regarding the link between grain-free diets and heart disease.

Grains and grain-related ingredients are commonly utilized as cost-effective sources of energy and nutrients in animal food, including pigs, birds, cats, dogs, and other animals [7,8]. In response to consumer demand for premium and natural ingredients, pet food manufacturers began marketing grain-free diets as a healthier and more natural option, and some pet caregivers believed that grains, particularly those containing gluten such as wheat, were potential allergens for pets [9]. Grain-free diets were marketed as a solution to address potential food sensitivities and allergies [10]. Based on the definition of grain-free diet, wheat, barley, rice, maize, sorghum, spelt, bulgar, farro, millet, oats, rye, malt, brewer’s yeast, wheat starch, triticale (a wheat/rye hybrid), and other grains, as well as grain-derived components, are not allowed to be included in grain-free diets [11]. However, some foods marketed as grain-free may still contain ingredients such as brewer’s yeast, or they may be produced in facilities that also process grains. These cases do not comply with the strict definition of a grain-free diet. Additionally, there is also a prevalent promotion of so-called “gluten-free” diets in the market. Gluten is a composite of gliadin (a prolamin) and glutenin [12]. Among these, gliadin is the primary gluten protein responsible for triggering gluten intolerance [13]. Gluten-free diets are formulated to exclude wheat, rye, barley, and their hybrids or to remove gluten using specific processing techniques.

In the past decade, grain-free diets have garnered plenty of attention. A survey on the purchase of grain-free dog food examined the consumption habits of dog caregivers in five countries: the United Kingdom, Germany, France, the United States, and Canada, as detailed in Table 1 [14]. This trend is primarily associated with the incidence of dog allergies and the eating and purchasing habits of caregivers, as illustrated in Table 2 [14]. Among them, individuals who responded “yes” to feeding their dog a specific diet due to a perceived food allergy were four times more likely to select “grain-free” than those who chose “no”. Furthermore, people who disagreed that grains should be part of a healthy diet were 1.6 times more likely to choose “grain-free” options than those who agreed. Simultaneously, regarding purchasing habits, it can be found that consumers are more inclined to obtain information and purchase dog food online. Moreover, the ingredient list has consistently been the most critical factor for consumers when selecting pet food [14,15,16]. Nevertheless, it is important to note that this is necessarily a valid way to select pet food. The nutrient profile, rather than the ingredient, should be the primary focus. In summary, consumers tend to use ingredients as a proxy for nutritional quality, and if they perceive certain ingredients as healthy, they are more likely to choose them for their pets. Importantly, grains, which are a prevalent plant-based ingredient in pet dry food and provide a cost-effective source of energy [17], provide essential nutrients and fiber, which are critical for animal growth and health [18]. Nevertheless, the growing consumer demand for grain-free diets has led to the replacement of traditionally used and generally well-tolerated grain ingredients. This change may pose unintended health risks to pets. As reported by the FDA, between 2014 and 2019, 524 cases of DCM in pets were documented, resulting in 124 fatalities [6]. Therefore, adopting a scientifically grounded and rational approach to evaluating pet food ingredients is essential for ensuring the healthy feeding of pets.

Currently, controversy persists surrounding grain-free pet food. This review investigates the key nutritional components of grain-free diets and carried out a comprehensive summary and review of the existing studies related to grain-free diets. It primarily examines the potential impacts of grain-free diets on pet health and nutrition. The article intends to offer scientific grounds for pet caregivers, veterinarians, researchers, and other pet professionals to understand grain-free pet food, facilitating more accurate and efficient pet feeding decisions.

## 2. Methods

We utilized Web of Science, PubMed, and Google Scholar as our primary search engines and databases. Additionally, we employed both backward and forward snowballing methods to identify more relevant citations within selected references. The final search was executed in June 2025. Our search strategy focused on the following keywords in the title, abstract, or keyword fields: grain-free, pet food, dog, cat, carbohydrate, DCM, nutrition, and health.

## 3. Alternatives for Grains in the Grain-Free Diets and Nutrient Composition Analysis

### 3.1. Grain Alternatives and Carbohydrate

The macronutrient balance in grain-free diets for pets can vary significantly depending on the ingredients and formulation of the diet [19]. Generally, grain-free diets aim to provide essential nutrients without relying on grains as a significant source of carbohydrates. Given that the grains are eliminated in the diet, alternative sources of carbohydrates are needed to provide energy and essential nutrients in the absence of conventional grains, as well as to facilitate the extrusion process in the production of pet dry food. Legumes and tubers are commonly incorporated as ingredients to provide a carbohydrate source. A comprehensive review of existing literature on the main components of commercial grain-free dry diets has found that the most common plant-based carbohydrates in these diets are derived from peas, potatoes, sweet potatoes, lentils, cassava, and chickpeas, in that order [20,21,22,23,24]. Among them, peas are widely regarded as ideal ingredients in human diets and pet food due to their high protein content, rich carbohydrates, dietary fiber, and various beneficial vitamins [25]. Despite their nutritional value, leguminous seeds contain anti-nutritional factors such as amylase inhibitors, trypsin inhibitors, and phytohemagglutinin, which may pose potential health risks [26,27]. Fortunately, these compounds can be effectively deactivated through heat treatment [28,29]. In dog food application research, heat-treated chickpeas or peas used as substitutes for commercial dog food have been proven to be safe. Nevertheless, whether they exert subtle effects on other physiological aspects, such as digestibility, still requires further investigation [30]. Additionally, the study found that adding peas might alter the fecal microbiome composition of healthy dogs [30]. Furthermore, compared with corn starch or traditional starch sources primarily composed of corn and rice, potato starch has been reported to significantly increase puffing, reduce the density of coarse-ground food, and enhance digestibility [31,32]. It also improves food palatability [31]. Similarly, a potato-based diet was found to affect the fecal microbiome of dogs, specifically manifested by an increased molar ratio of lactic acid, decreased fecal pH, and reduced ammonia levels [31,33]. However, even in the absence of grains, commercially prepared grain-free diets can exhibit varying levels of carbohydrates depending on the inclusion of legumes and tubers. Studies have shown that, on average, the carbohydrate (or nitrogen-free extract) content of grain-free diets has been lower than that of grain-inclusive diets. However, it should be noted that there is significant overlap between the two groups, and the carbohydrate content in commercial pet diets varies substantially among different manufacturers, geographical origins, and types of pet food [20,21,22,34,35]. In other words, grain-free diets should not all be considered equivalent to low-carbohydrate diets. Typically, a dry product labeled as low carbohydrate must contain less than 15 percent carbohydrate content [36].

Although cats and dogs do not have minimum dietary carbohydrate requirements [37,38], glucose, as the fundamental unit of carbohydrates, serves as the primary energy source for animal bodies and is physiologically essential. Meanwhile, numerous studies have demonstrated that cats and dogs can efficiently digest properly cooked and processed carbohydrates, with digestibility often exceeding 90% [39,40,41,42]. However, it is important to note that although carbohydrates can fulfill the energy requirements of cats, as obligate carnivores, felines possess limited metabolic capacity for carbohydrate processing. Specifically, they are unable to inhibit gluconeogenesis [43], and their glycolytic capability is relatively weak due to factors such as low glucokinase activity [44,45,46]. Additionally, carbohydrates offer certain health benefits for cats and dogs. For instance, fermentable carbohydrate sources can promote the fermentation of colonic bacteria, thereby supporting intestinal health [47,48]. It was found that adding fiber sources to the cat diet increased the concentration of butyrate in cat feces [49]. Butyrate promotes gluconeogenesis [50], thereby helping maintain the stability of blood sugar levels in cats. Nevertheless, it should be noted that excessive consumption of high-carbohydrate or high-fat diets may lead to elevated postprandial blood sugar levels and increase the risk of diabetes [51,52,53,54].

### 3.2. Protein and Fat

To simultaneously achieve grain-free characteristics and high nutritional levels, it is crucial to precisely regulate the inclusion of non-grain carbohydrate sources and appropriately adjust the protein and fat contents. Generally, grain-free diets tend to have higher protein and fat levels compared to grain-containing diets [19,21,22,32,34]. However, this trend is not universal. Some grain-free diets exhibit protein or fat levels that are comparable to or even lower than those in grain-containing diets [34,55,56]. Nonetheless, excessive protein content in pet food may potentially affect dogs adversely. While earlier studies have indicated that consuming substantial amounts of high-quality protein does not impair kidney function and that older dogs do not necessarily require reduced protein intake [57,58], recent research has revealed that high-protein diets might negatively impact intestinal digestion [59,60]. In addition, fat, as the macronutrient with the highest energy density, provides more than twice the energy per gram compared to protein or carbohydrates [61]. In grain-free diets, fat content is often increased to a moderate or high level to supply energy, enhance palatability, and support skin and coat health [62,63]. A deficiency in dietary fat and essential fatty acids may lead to weight loss and compromised skin and hair quality [64], whereas excessive fat intake may contribute to obesity development [65]. In summary, both excessive and insufficient protein and fat levels can influence animal health.

The European Pet Food Industry Federation (FEDIAF) established a good nutritional guideline for European pet food manufacturers [37,38], ensuring the production of safe pet food. Regardless of whether the pet food is grain-free or not, the protein and fat contents of the studied commercial pet foods generally meet FEDIAF’s minimum nutritional requirements [22,34]. Furthermore, Debraekeleer et al. [66,67] proposed recommended ranges of protein and fat for dogs at different life stages (on a dry matter basis): growing puppies should have crude protein (CP) levels between 22% and 32%, and fat levels between 10% and 25%; adult dogs should have CP levels ranging from 15% to 30%, and fat levels from 10% to 20%. Using this as a reference, all diets analyzed have fat and metabolizable energy contents within the recommended ranges, but some grain-free diets exceed the suggested maximum CP levels [34]. Table 3 summarizes the nutrient composition of commercial grain-free diets from existing studies, including crude protein, crude fat, crude fiber, crude ash, nitrogen-free extract, and metabolizable energy content, among others.

### 3.3. Amino Acids and Other Micronutrients

In addition to meeting their primary nutritional requirements, companion animals require an adequate supply of essential micronutrients in their diets, such as minerals and amino acids, to achieve a healthy and balanced nutritional profile. Studies have shown that the essential amino acid content in grain-free dog food is generally higher than that in grain-containing dog food, which may be attributed to the higher proportion of animal-based ingredients in the formulation [34]. However, it was observed that the phenylalanine content in a few single-grain-free diets fell below the recommended threshold, while lysine levels exceeded the recommended range, indicating the need for careful attention to amino acid balance [34]. Regarding trace elements, grain-free diets exhibit significantly higher concentrations of P, K, Na, Fe, Zn, Mn, and Cu compared to grain-containing diets. Nevertheless, some products have excessive levels of Fe, Mn, and Zn, especially in grain-free foods made from insect protein, where Mn may exceed the FEDIAF legal limit [37,38]. Although no heavy metals were detected in any of the samples, some grain-free and grain-containing diets exhibited abnormal Ca:P ratios, with approximately 27% of the samples being unsuitable, potentially impacting bone health [24]. Overall, grain-free diets offer advantages in terms of nutrient density for certain trace elements but require careful raw material selection and formula optimization to avoid issues of excessive element content and imbalanced proportions.

To summarize, it is important to highlight that, irrespective of whether pet food contains grains, its specific nutritional composition may vary significantly due to differences in formula design and brand variations [19,20,21,22,34,35]. While grain-free diets may suit certain pets’ needs appropriately due to their special ingredients, it is vital to emphasize that the overall nutritional quality of the diet holds greater significance than simply excluding grains.

## 4. Implications of Grain-Free Diets on Pet Health and Welfare

**Figure 1:**
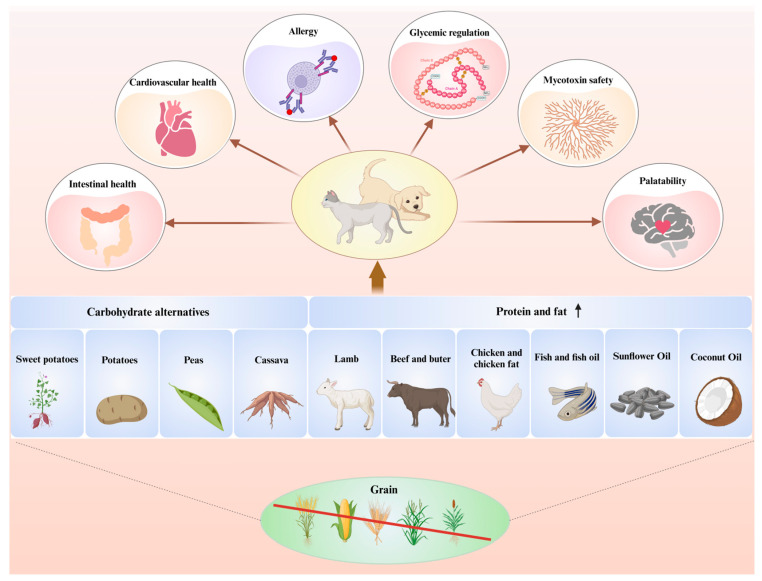
Implications of feeding grain-free diets on health and welfare in dogs and cats. Current experimental evidence indicates that diets devoid of grains can exert potential benefits and risks on the health and well-being of dogs and cats, encompassing aspects such as gastrointestinal function, cardiovascular performance, allergic responses, blood glucose regulation, mycotoxin safety, and gustatory preferences. The figure was created in BioRender.com.

Currently, despite the certain share of grain-free pet diet in the market, controversies persist regarding the nutritional impact and potential health effects of these diets on pets, such as DCM. In this section, we present recent advancements in research concerning the effects of a grain-free diet on various aspects of canine and feline health including gastrointestinal health, cardiovascular health, allergy, glycemic regulation, mycotoxin safety, and palatability (Figure 1), which elucidate the relationship between grain-free diets and dogs’ as well as cats’ overall wellbeing. By fully comprehending both the potential benefits and risks associated with adopting a grain-free diet, we can make a more scientific decision and provide our pets with more assurance for their health.

### 4.1. Gastrointestinal Health

Grain-free diets for pets have garnered popularity, with numerous pet caregivers opting for them based on diverse beliefs regarding the nutritional requirements of dogs and cats. However, it is imperative to consider pets’ digestive physiology when assessing the suitability of a grain-free diet. The gastrointestinal well-being of pets is intricately intertwined with their overall health, and dietary choices play a pivotal role in maintaining an optimal digestive system.

Digestibility is a critical factor in pet nutrition research, as it directly influences the intake and absorption of nutrients essential for animal health [68]. In recent years, studies on the effects of grain-free diets on digestibility in dogs have revealed multifaceted outcomes. Table 4 summarizes findings from current research comparing grain-inclusive and grain-free pet foods in terms of their impact on dog digestibility. Digestibility is influenced by various factors, including not only the presence or absence of grains in the food but also other ingredients, processing characteristics, and individual pet conditions [19,69,70]. Studies indicated that high-animal-protein grain-free diets, in which 70% of the dietary protein is derived from animal sources, exhibited superior comprehensive digestibility, with significantly higher dry matter digestibility, organic matter digestibility, and CP digestibility compared to other formulations in which 45% of the dietary protein originates from animal sources [32,70]. However, it is important to note that higher protein content does not necessarily equate to better digestibility. Excessive protein intake may reduce beneficial gut bacteria such as lactic acid bacteria and *Enterococcus* spp., leading to increased ammonia and carcinogenic bioamine production, damage to intestinal villi structure, and ultimately reduced digestibility [59,60]. On the other hand, grain-free diets generally perform exceptionally well in terms of fat digestibility, which may be attributed to the higher fat content in these formulations [19,32,69,70]. Additionally, the findings on fiber digestibility of grain-free and grain-inclusive diets show inconsistency across different studies [19,69,70,71], and this inconsistency may stem from differences in fiber type, specifically the ratio of soluble to insoluble fiber across formulations. For instance, legumes such as peas contain higher concentrations of soluble fiber compared to grains, resulting in generally higher dietary fiber digestibility than diets based on other carbohydrate sources [41,72]. In contrast, cassava flour, another common grain-free ingredient, has relatively low soluble fiber content and consequently exhibits lower dietary fiber digestibility [41,72].

Fiber plays a significant role in gastrointestinal health and can be categorized into soluble dietary fiber and insoluble dietary fiber based on solubility. Soluble dietary fiber is fermented by colonic bacteria, promoting the production of metabolic products such as short-chain fatty acids (SCFAs) and contributing significantly to maintaining intestinal ecological balance [48]. Although insoluble fiber is not easily fermented by the large intestine, it helps maintain normal intestinal transit by promoting intestinal peristalsis [73]. Clark et al. reported that feeding grain-free diets increased SCFA levels in dog feces, potentially attributed to the rapid fermentation characteristics of pea fiber as a soluble dietary fiber in the intestine [70]. Additionally, resistant starch, which is difficult to digest and instead fermented by gut microbiota, exhibits properties similar to those of soluble dietary fiber. Potato fiber, primarily consisting of resistant starch and easily digestible starch, has been shown in studies to increase the concentrations of three SCFAs (acetate, propionate, and butyrate) in dogs’ diets, as well as enhance the proportion of *Faecalibacterium* spp. in the microbiota, highlighting their potential prebiotic properties [74]. While SCFAs contribute to improved intestinal health and reduced inflammation [75], excessive production may draw water and sodium into the intestinal lumen due to its osmotic effect, thereby increasing fecal water content [76]. Furthermore, multiple factors collectively influence fecal consistency, including crude fiber levels, fiber types, the presence of anti-nutrient factors, and crude ash content [77,78,79,80]. Some studies indicate that dogs fed grain-free diets exhibited lower dry matter content and softer fecal texture compared to those on grain-inclusive diets [69,70,71]. Nevertheless, fecal scores across all dietary groups remained within the ideal range [19,32,69,70,71].

In terms of gut microbiology, it was found that grain-free diets with low animal protein (including legumes) can increase the abundance of *Lactobacillaceae*, *Veillonellaceae*, and *Bifidobacterium* sp. However, the concurrent reduction in microbial α-diversity indicates a trend toward homogenization of the microbial community, necessitating further long-term studies to comprehensively assess health implications [70]. Additionally, low-animal-protein grain-free diets demonstrated decreased fecal concentrations of ammonia, indole, and secondary bile acids but elevated levels of total SCFAs and primary bile acids [70]. Conversely, high-protein grain-free diets may elevate fecal ammonia and indole levels [32], implying potential risks associated with protein fermentation. Collectively, grain-free diets modulate gut microbiota through adjustments in fiber and protein sources, with combined effects varying by breed and dietary formulation. At the same time, considering the critical role of the intestinal microbiota in metabolism and its association with gastrointestinal disorders and other diseases, it is imperative to evaluate microbiota and microbiota-derived metabolites in animals fed with grain-free diets for a long-term period, and further research is warranted in this area.

### 4.2. Cardiovascular Health

Numerous alternative ingredients, such as beans and potatoes, have been introduced into grain-free diets as alternative sources of carbohydrate. However, comprehensive studies on the long-term safety of these novel components are yet to be completed. It has been observed that grain-free diets may potentially impact the cardiovascular health of pets. In a report released by the FDA [6], intake of grain-free diets, particularly those high in beans, potatoes, or sweet potatoes, may be associated with increased risk of canine DCM. Its symptoms are characterized by dysfunctional expansion and contraction of the left ventricle, and the most commonly reported comorbidity was low blood taurine levels [81,82]. It is important to note that while blood taurine serves as a practical biomarker, its levels may not consistently reflect tissue-specific taurine status, particularly in the heart. The etiology of DCM primarily involves genetic factors, immune-mediated diseases, viral infections, toxins, and nutritional deficiencies [83]. This report linked nutrients with the development of DCM in several breeds of dogs, including those without genetic predisposition to DCM, raising further concerns about the potential safety of grain-free diets. Bakke et al. showed that feeding a grain-free diet with a high legume content may lead to erythropoiesis and hyperphosphatemia, as well as disturbances in taurine status, which showed a commonality with dogs suspected of DCM [84]. Chloe et al. demonstrated an association between a pea-based diet and the development of DCM in dogs, whereas no such related symptoms were observed in dogs fed a lentil-based diet [85]. In a separate study, the occurrence of DCM in canines fed a diet devoid of grains was effectively reversed upon transitioning to a diet containing grains [86]. In addition to signs of DCM, diet-related DCM showed subclinical abnormalities in heart size and function, as well as elevated cardiac biomarkers [87,88,89,90,91,92], which were reversed following a change the diet, and even exhibited increased longevity [6,81,86,93,94,95,96,97]. Interestingly, several studies have reported that grain-free diets are not necessarily associated with the development of DCM. Leach et al. established four dietary groups (high-protein grain-free group, low-protein grain-free group, high-protein grain-inclusive group, and low-protein grain-inclusive group) fed to Beagles and Mixed Retrievers for 30 weeks. The study demonstrated that none of the four diets induced a detectable DCM phenotype [98]. Donadelli et al. observed that Labradors fed a grain-free diet for 26 weeks had no effect on plasma taurine and general health indicators [99]. Similar results were reported in dogs fed pulse-based grain-free diets [100,101]. However, caution should be exercised when applying these research findings, as the studies cited were all short-term trials involving different dog breeds and different formula qualities. It is currently difficult to clearly establish a causal relationship between grain-free diets and DCM. This renders the long-term effects of different dog breeds, complex and varied pet food formulations, or other processing approaches (such as canned or raw food diets) on canine health, an area where questions still remain.

Furthermore, although the majority of cases reported have involved dogs, there have been over 20 suspected instances of DCM in cats as per data of July 2020 [6,81]. Currently, there is limited research investigating the association between grain-free diets and DCM in cats. In a retrospective multicenter study of 37 cats with DCM, 38% of the cats fed high-pulse diets, and taurine content equaled to or greater than the requirement [102]. Moreover, Shelby et al. found that although there was no significant association between high-pulse diets and heart size, function, or biomarkers in cats, a significant negative correlation was observed between the duration of eating high-pulse diets and left ventricular wall thickness [103]. A relationship might be validated by the inclusion of more cats in the experimental study. In summary, a grain-free diet may influence the onset of DCM. Nevertheless, the causal relationship between the two remains unclear at present. However, it is important to emphasize that the fundamental nutritional compositions of dog and cat food are not directly comparable. Unlike dog food, cat food typically contains higher levels of fat and protein, as well as added taurine, which may help mitigate the risk of health issues in cats. Furthermore, some cats may supplement their diet by consuming animal matter through hunting. Current data are insufficient to establish definitive conclusions linking grain-free diets or specific dietary components to DCM, and further research is warranted.

### 4.3. Allergy Response

Allergic response refers to a symptomatic response resulting from an antigen–antibody interaction triggered by external stimuli. Common pet allergens encompass inhalation allergens (such as pollen, dust mites, and animal dander), contact allergens (including fleas, bacteria, and molds), injection allergens (like penicillin and xenoserum), food allergens, and autologous tissue antigens. According to surveys, many pet caregivers appear to hold the belief that eliminating common grain ingredients reduces exposure to potential allergens [14]. However, it should be noted that food-induced allergies are just one of several causes of allergies and, in fact, occur relatively infrequently. Food allergy refers to an immune-mediated response triggered by a normal food or ingredient [104]. The survey shows that allergies related to food account for less than 1% of dog skin disorders or 10% of allergic skin disorders [9]. Moreover, in a study involving 128 cats with scratching or gastrointestinal symptoms, only 17% were diagnosed with food allergies [105]. The majority of allergens in this allergy consist primarily of proteins or glycoproteins [9]. Since an allergy represents an abnormal or inappropriate response by the immune system towards normal proteins, any protein-containing food or ingredient may potentially trigger an allergic reaction.

Combining past cases of food allergies, the most prevalent food allergens in dogs include beef, dairy products, chicken, and wheat [104,106,107], while in cats they include dairy products, beef, and fish [104,106,107]. Additionally, among plant-derived ingredients, corn, soybean, rice, and barley, although not the main allergens, may also cause allergic reactions in some cats and dogs [108,109,110,111,112,113,114,115]. Furthermore, in recent years, several novel methods for detecting allergens have been developed to assist in identifying allergens in pets. Dodds analyzed over 1000 saliva samples from dogs and cats using saliva IgA/IgM tests (threshold ≥ 11.5 U/mL). The study revealed that the likelihood of a high IgA/IgM response in dog saliva to plant-based foods was as follows: corn (12%), wheat (5%), peanut (4%), and rice (3%) [116]. In feline subjects, plant-derived components eliciting mean IgA/IgM responses above 11.5 U/mL included rice (25.9 U/mL), millet (24.3 U/mL), potato (23.2 U/mL), quinoa (19 U/mL), sweet potato (17.1 U/mL), oats (15.5 U/mL), soybean (13.4 U/mL), corn (12.67 U/mL), and peanuts (12.2 U/mL). Comparatively lower mean salivary IgA/IgM responses were observed in cats for wheat, barley, and lentils [117]. Serological tests demonstrated that the allergic rates of corn and potato in dogs with atopic dermatitis were 38.3% and 28.7%, respectively [118]. Prick tests indicated that among 25 dogs with a history of skin issues, 20 reacted to wheat and 11 to soy [119]. However, it is important to note that these detection methods lack sufficient specificity, and their accuracy requires further validation. The exclusion diet method remains the gold standard for detecting dietary allergies in pets [120,121]. On the other hand, some studies have shown that diets based on potatoes, rice, or peas can serve as hypoallergenic options, demonstrating potential applications in alleviating adverse food reactions [122,123,124,125,126,127].

It is worth mentioning that the sensitivity caused by grain gluten may lead to multi-system disorders involving the gastrointestinal, nervous, and skin systems, such as paroxysmal gluten-sensitive movement disorder and gluten-sensitive enteropathy. Canine paroxysmal dyskinesia (also known as canine epileptoid spasticity syndrome) is a neurological condition characterized by sudden, involuntary movements. Recent studies have found that some cases of this disease are significantly linked to gluten diet sensitivity, particularly in border terriers [128,129,130,131]. Lowrie et al. discovered that serum anti-transglutaminase 2 (TG2 IgA) and anti-glutenin (AGA IgG) antibody levels were significantly elevated in affected dogs, suggesting an immune-mediated gluten reaction [129]. After strictly adhering to a gluten-free diet, clinical symptoms (such as limb dystonia and tremors) and serum antibody levels improved significantly in all dogs, and in some cases, symptoms resolved completely. Symptom recurrence was associated with gluten reintroduction, confirming the central role of dietary management [128,129]. Moreover, the case report further demonstrated that certain paroxysmal gluten-sensitive movement disorders were accompanied by gastrointestinal inflammation, skin allergy, and other symptoms, while a gluten-free diet not only improved movement disorders but also alleviated intestinal and skin symptoms, suggesting that gluten induces a systemic inflammatory response through immune mechanisms [130,131]. Additionally, some studies have shown that in other dog breeds, certain dogs with paroxysmal dyskinesia tested positive for gluten sensitivity [132], and switching to a gluten-free diet significantly improved their symptoms [132,133,134,135]. On the other hand, within Irish Setter families, a significantly higher prevalence of gluten-sensitive bowel disease has been observed [136]. The primary risk factors are genetic predisposition and consumption of a wheat gluten-containing diet [136]. Specific manifestations include abnormal intestinal immune responses, leading to diarrhea, weight loss, malnutrition, and other symptoms [137]. Studies have shown that the concentration of anti-glutenin antibodies in affected dogs is significantly reduced [138], and adopting a gluten-free diet effectively alleviates gluten-sensitive bowel diseases [139,140].

In conclusion, while many pet caregivers opt for a grain-free diet due to concerns about food allergies, it is important to note that both grain-inclusive and grain-free diets may trigger pet allergies, with risks varying by individual.

### 4.4. Glycemic Management

At present, extensive research has demonstrated that altering dietary carbohydrate sources can influence postprandial blood glucose concentrations in dogs and cats [41,42,52,141,142,143,144,145,146,147,148,149]. Studies on canine nutrition have revealed that diets containing sorghum, lentils, or peas exhibit greater advantages in delaying and prolonging blood glucose and insulin responses compared to diets with corn, brewer’s rice, or cassava flour [41]. This conclusion was subsequently corroborated by further studies. Specifically, the use of grain-free diets based on legumes notably delayed postprandial glucose and insulin responses, with a mean glycemic index (GI) of 41, which is lower than the GI of traditional grain diets (e.g., wheat and corn) as well as whole-grain diets (e.g., brown rice, barley, oats, and rye). According to the human GI classification standard, this grain-free formula qualifies as a low-GI food (GI ≤ 55) [147]. Similar findings were confirmed in another comparative study [141]. This effect may be attributed to the relatively high amylose content, resistant starches, and dietary fiber content. These diets exhibit slower rates of digestion [150,151]. Additionally, sweet potato-based grain-free diets demonstrated significant moderating effects, exhibiting lower postprandial glucose and insulin area under the curve (AUC ≤ 360 min) compared to pea-based and grain-based diets [152].

In contrast to dogs, feline carbohydrate metabolism exhibits distinct species-specific differences. Current studies indicate that the source and content of starch have limited impacts on postprandial glucose and insulin responses in cats [42,148,149], potentially due to their carnivorous biological traits [153]. The low activity of salivary amylase and intestinal amylase in cats might contribute to reduced sensitivity to metabolic responses from various carbohydrate sources [154]. In a comparative analysis of six carbohydrate sources (brewer’s rice, corn, cassava flour, sorghum, peas, and lentils), a corn-based diet elicited stronger glycemic and insulin responses, whereas a lentil-based diet exhibited superior metabolic homeostasis [42]. Subsequent comparative studies on tubers (potatoes, cassava, sweet potatoes) and cereals (rice, wheat) revealed no statistically significant differences in blood glucose responses among the five groups [148]. However, diets containing cassava or sweet potatoes showed potential benefits in controlling blood glucose and lipid levels [148].

In summary, diets rich in legumes or sweet potatoes offer better control over blood sugar levels and insulin sensitivity. Furthermore, physiological states like diabetes, obesity, pregnancy, stress, infection, and cancer, as well as aging, can all influence glycemic control [155]. At the same time, hyperglycemic reactions are not only a consequence of metabolic disorders but also recognized risk factors for their development. Chronic consumption of high-glycemic-index foods may induce insulin resistance, further exacerbating these symptoms [155]. In these instances, the implementation of a dietary regimen that optimizes postprandial glucose and insulin responses (e.g., by feeding a grain-free diet), thereby enhancing glycemic control and promoting pet well-being, can be advantageous [156,157]. On the other hand, elevated postprandial blood glucose levels are recognized as a well-established risk factor for cardiovascular disease in both healthy and diabetic individuals, potentially initiating a cascade of detrimental events within the cardiovascular system [158,159,160,161]. Scientific evidence has demonstrated that incorporating low GI foods into the diet can effectively enhance indicators associated with cardiovascular disease risk reduction [141,162,163,164,165,166,167]. Notably, besides starch sources, fat levels can also impact postprandial blood glucose in cats and dogs. Research shows that low-fat diets are more advantageous for weight management, inflammation reduction, and blood sugar regulation in cats [168]. This suggests that when selecting a grain-free diet, it is important to be mindful of its fat content.

### 4.5. Mycotoxin Security

Mycotoxins, which are abiotic hazards produced by certain fungi, pose a significant threat to human and animal health through various cytotoxic mechanisms [169]. These mycotoxins, known as naturally occurring toxins, can be found in human food and animal feed, with cereals and their products being the primary sources. Common mycotoxins include aflatoxin, fumonisins, ochratoxin A, and zearalenone. The effects of mycotoxins on animals vary depending on the type, concentration, and duration of exposure. It is worth noting that even long-term exposure to low concentrations of mycotoxins can be harmful as they can have a negative impact on effective immune responses, leading to chronic diseases such as liver and kidney fibrosis or diseases caused by compromised immunity [170,171]. Furthermore, a study demonstrated that mixtures of mycotoxins extracted from different dog diets (including ochratoxin A, zearalenone, aflatoxin B1, aflatoxin B2, fumonisins B1, and fumonisins B2) exhibited immunotoxicity towards canine peripheral blood mononuclear cells [169].

In recent years, the growing focus on animal welfare has underscored the potential risk of mycotoxins in companion animals. Although regulatory agencies for animal feed and food safety, such as the UK Food Standards Agency (FSA) [172] and the European Commission (EC) [173], have established maximum permissible concentrations for certain mycotoxins in food, these standards are typically defined for human food and livestock feed. They have not yet established scientifically grounded limits specifically and exclusively for pet food end products. Limits for mycotoxin levels in pet food are typically extrapolated from those set for livestock [174]. Furthermore, due to mycotoxins being resistant to heating and chemical inactivation in downstream processing steps, preventing contamination by mycotoxins poses significant challenges [175,176]. Companion animals are often maintained on a monotonous grain-based diet for extended periods, thereby elevating their potential exposure to pet food contaminated with either single or multiple mycotoxins [174].

Due to the exclusion of grains from the grain-free diet, the concentrations of total mycotoxins, including total aflatoxin, ochratoxin, fumonisins, and zearalenone, in grain-free diets were lower than those in other diets [69]. In a separate study, low levels of *Fusarium*-derived mycotoxin contamination were detected in dry dog diets containing grains, but no mycotoxin contamination was detected in grain-free diets [177]. In conclusion, grain-free diets can reduce mycotoxin contamination in pet food and reduce potentially deleterious effects on animals. Although the concentrations of mycotoxins in grain-containing diets are far below the amounts considered to be acutely toxic, these data supported the possibility that feeding grain-containing diets can lead to chronic exposure to various mycotoxins. The long-term effects of low levels of mycotoxin contamination on dogs are still unknown and require further research.

### 4.6. Palatability Effects

While traditional livestock feed prioritizes production indexes such as feed conversion rate and daily gain, pet food differs in that palatability serves as the primary criterion for measuring product performance [178]. Palatability refers to a certain food’s ability to stimulate a pet’s taste, vision, and touch through its physical and chemical properties during consumption, resulting in favorable or unfavorable reactions. Guazzelli et al. discovered that dogs exhibited a preference for grain-free diets over those containing grains [69]. This finding was further supported by Kahraman et al., who reported that 88.44% of dogs preferred grain-free diets to those with grains included [71]. Therefore, certain components of these diets may possess greater appeal and palatability to dogs. The subsequent investigation revealed that canines exhibited a preference for a diet characterized by an elevated content of potato starch [31]. Potato tuber tissues release 5′-ribonucleotides (such as IMP and GMP) during cooking as RNA degrades, which are precursors of umami compounds and serve as flavor enhancers [179]. Consequently, tissues, one of the primary constituents in grain-free diets, may significantly influence palatability. However, research also demonstrated that the concentration of total volatile compounds in samples containing grains was higher than in grain-free samples, resulting in reduced aroma perception in the latter, primarily due to elevated concentrations of aldehydes present in grain-added samples [180]. In summary, compared to diets containing grains, grain-free diets may exhibit distinct tastes and textures. Each pet exhibits distinct characteristics, leading to considerable variation in their food preferences and responses to dietary changes. When considering or implementing any dietary modifications, it is imperative to closely observe your pet’s reaction to the new diet.

## 5. Conclusions and Future Directions

Grain-free diets, defined as pet foods that exclude traditional cereal grains (e.g., wheat, barley, rice, maize) and their derivatives, have been gradually gaining popularity due to the market’s emphasis on the natural, hypoallergenic, and health-enhancing attributes, as well as common misconceptions among consumers (such as the belief that cats and dogs do not eat plant-based materials). However, scientific evaluations indicate that the benefits of grain-free diets coexist with potential risks. Advantages include the effective elimination of allergens for pets with grain or gluten sensitivities, the potential reduction in mycotoxin exposure through the removal of grain ingredients, enhanced blood sugar control via formulations rich in legumes or tubers, and increased palatability achieved by incorporating alternative carbohydrate sources. However, it is crucial to highlight that grain-free diets, particularly those high in legumes, have been potentially linked to canine dilated cardiomyopathy. Furthermore, some grain-free foods may impose an increased metabolic burden due to their elevated protein or fat content. While there are compositional differences between grain-free and grain-containing diets, most products in both categories meet fundamental nutritional balance requirements. Equally, neither type can ensure a remarkable reduction in the risk of allergies. Current evidence underscores that grain-free diets are neither universally superior nor unalterably detrimental. Their appropriateness depends on individualized factors, including confirmed allergies and species-specific needs. When selecting pet food, priority should be placed on a nutritionally balanced diet that aligns with pets’ physiological requirements, rather than being driven by market trends. Ultimately, a diet’s capacity to fulfill comprehensive nutritional standards, whether grain-free or grain-inclusive, remains the cornerstone of companion animal health.

## Figures and Tables

**Table 1:** Percentage of respondents in each country who consider “grain-free” when selecting pet food.

Country	Percentage of Respondents in Each Country Who Seek “Grain-Free” Options in Pet Food	Reference
Germany	30.0%	[14]
UK	19.6%	
Canada	21.8%	
America	27.4%	
France	8.0%	

**Table 2:** Multinomial logistic regression analyses of allergy, diet, and purchasing habits models were used to determine which variables predicted consumer choice of grain-free dog food.

Multinomial Logistic Regression	Variable		*p*-Value	Odds Ratio	Reference
Allergy Model	Do you feed your dog a specific diet because you believe your dog has a food allergy?	Yes	<0.0001	4.01	[14]
		No	-	-	
	Allergy Symptoms	One	0.422	1.094	
		Two or more	0.047	1.321	
		None	-	-	
	Do you feed your dog a specific diet because your dog has been diagnosed by a veterinarian with a food allergy?	Yes	0.041	0.633	
		No	-	-	
	When choosing a pet food, I look for…	Sensitive skin/stomach	<0.0001	1.692	
		Limited ingredient diet	<0.0001	3.019	
		Exotic protein	<0.0001	1.904	
Diet Model	I try to eat grains as part of a healthy diet	0–4 (Disagree)	0.003	1.646	
		5 (Neutral)	0.003	1.439	
		6–10 (Agree)	-	-	
	When choosing a pet food, I look for (poultry, beef, fish, pork, exotic protein, organic/natural, vegetarian/vegan, limited ingredient diet)	1–3 options selected	0.019	1.625	
		4 or more options selected	0.01	1.759	
		No options selected	-	-	
	When choosing a pet food, I look for…	No fillers ^1^	<0.0001	2.621	
		No by-products	<0.0001	1.553	
	Other food items (dog treats, table scraps, fruits/veggies, other) given on a daily basis	One option selected	0.619	1.056	
		Two or more options selected	0.006	1.439	
		No options selected	-	-	
Purchasing Habits Model	I purposely rotate my dog’s dry food to provide variety	True	0.021	0.799	
		False	-	-	
	Where do you get your information about dog food from?	Veterinarian	0.232	0.891	
		Online	<0.0001	1.571	
		Pet store staff	0.006	1.337	
	Where do you purchase your pet food from?	Vet clinic	0.002	2.197	
		Pet specialty store	<0.0001	1.572	
		Online	<0.0001	2.371	
		Other	0.156	1.355	
		Grocery store	-	-	

The content of this table is derived from Sydney Banton et al., 2021 [14], who extracted and integrated the original content. The *p*-values < 0.05 indicate statistically significant predictors of grain-free diet selection. Odds ratios represent the likelihood of choosing grain-free options when exposed to specific variables versus baseline conditions. ^1^ Fillers refer to components perceived by consumers as being added to pet food to reduce production costs, such as grains and pomaces, which are considered low in nutritional value. Nevertheless, it is important to note that this perception is rooted in consumers’ preconceived notions. In reality, many of these components possess significant nutritional value.

**Table 3:** Nutrient composition (g/100 g dry matter) and metabolizable energy (kcal/100 g dry matter) of analyzed commercial grain-free diets.

Food Group		*n*	CP	EE	CF	TDF	CA	NFE	Starch	ME	Reference
Dog food	Grain-Inclusive	12	30.85	14.25	6.80	-	7.63	40.47	-	379.28	[21]
	Grain-Free	23	31.78	14.91	7.12	-	8.89	37.31	-	375.93	
Dog food	Grain-Inclusive	19	25.53	10.75	6.12	-	6.63	44.68	-	369.50	[22]
	Grain-Free	17	31.14	15.13	8.57	-	7.39	31.50	-	369.40	
Dog food	Grain-Inclusive	13	25.75	11.31	6.31		6.71	43.68		379.60	[24]
	Grain-Free	17	30.69	14.71	8.40		7.45	33.20		387.93	
Adult and senior dog food	Low price ^1^, Grain-Inclusive	10	23.75	14.42	2.20	-	6.64	53.56	-	384.74	[34]
	Medium price, Grain-Inclusive	10	27.02	13.02	1.44	-	6.69	51.83	-	413.09	
	High price ^1^, Grain-Free	10	32.50	14.99	2.30	-	8.60	41.83	-	412.34	
Puppy food	Low price ^1^, Grain-Inclusive	10	30.14	17.83	1.64	-	7.05	43.35	-	403.66	
	Medium price, Grain-Inclusive	10	31.42	16.26	1.47	-	7.73	43.13	-	426.18	
	High price ^1^, Grain-Free	10	38.54	16.50	1.71	-	8.44	34.80	-	425.75	
Dog food	Grain-Inclusive	14	29.08	11.25	5.72	-	7.70	-	37.93	359.80	[19]
	Grain-Free	7	37.00	14.08	4.12	-	8.32	-	28.95	377.39	
Dog food	Grain-Inclusive	5	28.24	14.48	-	8.38	7.65	35.06	28.36	-	[35]
	Grain-Free	5	26.62	14.94	-	10.27	7.67	34.26	27.52	-	
Cat food	Grain-Inclusive	5	34.96	13.62	-	10.36	7.05	28.68	23.26	-	
	Grain-Free	5	37.96	14.44	-	9.12	7.62	25.66	20.50	-	
FEDIAF recommended minimum level											[37,38]
Dog food	Adult	-	18.00	5.50	-	-	-	-	-	-	
	Early Growth (<14 weeks)	-	25.00	8.50							
	Late Growth (≥14 weeks)	-	20.00	8.50	-	-	-	-	-	-	
Cat food	Adult	-	25.00	9.90	-	-	-	-	-	-	
	Growth	-	28.00	9.00	-	-	-	-	-	-	

n = sample size for each group; CP = crude protein; EE = ether extract or crude fat; CF = crude fiber; TDF = total dietary fiber; CA = crude ash; NFE = nitrogen-free extract; ME = metabolizable energy. The units of nutritional composition are expressed as g/100 g dry matter, which is equivalent to a percentage (%). Metabolic energy is expressed in kcal/100 g dry matter. ^1^ A price of less than 30 euros for a 12 kg pack is classified as low. A price ranging from 30 to 45 euros for a 12 kg pack is considered medium. A price exceeding 45 euros for a 12 kg pack is categorized as high [34].

**Table 4:** The evaluation of apparent nutrient digestibility of foods in the grain-inclusive and grain-free groups (%).

Breed of Dog	Food Group	DMD (%)	OMD (%)	CPD (%)	EED (%)	CFD (%)	TDFD (%)	Reference
Golden retriever	Grain-Inclusive	80.56	83.91	80.90	95.14 ^b^	42.70 ^a^	-	[20]
	Grain-Free	77.41	81.29	80.22	96.96 ^a^	23.83 ^b^	-	
Golden retriever	Grain-Inclusive	80.93	84.43	78.03	97.35	61.70 ^a^	-	[72]
	Grain-Free	80.65	80.65	78.77	96.74	53.23 ^b^	-	
Beagles	Grain-Inclusive	85.80	87.70	88.10	93.10	-	39.30 ^b^	[70]
	Grain-Free	85.80	87.00	87.20	93.60	-	51.80 ^a^	
Labrador retrievers	Grain-Inclusive	79.83	84.94	77.20 ^b^	90.67 ^b^	-	-	[33]
	Grain-Free	79.57	87.29	85.30 ^a^	96.62 ^a^	-	-	
Beagles	LP-Grain-Inclusive	81.80 ^c^	85.70 ^c^	82.50 ^b^	90.70 ^c^	-	48.00 ^c^	[71]
	LP-Grain-Free	85.60 ^abc^	88.00 ^bc^	86.70 ^ab^	95.20 ^b^	-	55.40 ^bc^	
	HP-Grain-Inclusive	85.30 ^bc^	88.20 ^ab^	86.10 ^ab^	93.60 ^b^	-	54.90 ^c^	
	HP-Grain-Free	89.40 ^ab^	91.10 ^ab^	88.20 ^a^	96.10 ^ab^	-	70.00 ^a^	
Mixed-breed hounds	LP-Grain-Inclusive	89.10 ^ab^	91.70 ^ab^	89.70 ^a^	94.60 ^b^	-	70.60 ^a^	
	LP-Grain-Free	86.70 ^abc^	89.10 ^ab^	87.20 ^ab^	95.80 ^ab^	-	61.30 ^abc^	
	HP-Grain-Inclusive	89.40 ^ab^	91.60 ^ab^	90.50 ^a^	95.50 ^b^	-	69.40 ^ab^	
	HP-Grain-Free	90.40 ^a^	92.00 ^a^	90.30 ^a^	96.30 ^a^	-	73.50 ^a^	

DMD = dry matter digestibility; OMD = organic matter digestibility; CPD = crude protein digestibility; EED = ether extract digestibility; CFD = crude fiber digestibility; TDFD = total dietary fiber digestibility. Superscripts with different letters in a column and within the same study represent statistically significant differences (*p* < 0.05).

## Data Availability

Not applicable.

## Abbreviations

The following abbreviations are used in this manuscript:

CP	Crude protein
DCM	Dilated cardiomyopathy
EC	European Commission
FDA	Food and Drug Administration
FEDIAF	European Pet Food Industry Federation
FSA	Food Standards Agency
GI	Glycemic index
SCFAs	Short-chain fatty acids
